# A Compassion-Focused Approach to Support Parents After Preterm Birth

**DOI:** 10.1007/s10567-025-00549-z

**Published:** 2025-10-06

**Authors:** Teagan M. Lloyd-Collins, Grace C. Fitzallen, James N. Kirby

**Affiliations:** 1 School of Psychology, The University of Queensland, Brisbane, Australia; 2 Compassionate Mind Research Group, Brisbane, Australia; 3 School of Psychology, College of Health and Medicine, University of Tasmania, Launceston, Australia

**Keywords:** Preterm birth, NICU, Parents, Postpartum depression, Anxiety, Posttraumatic stress disorder, Self-criticism, Shame, Compassion-focused therapy

## Abstract

Preterm birth remains a global health challenge with significant implications for neonatal outcomes and parental mental health. This paper explores the complex psychological experiences and intrapersonal processes of parents after preterm birth, highlighting their heightened risk for mental health difficulties such as postpartum depression, anxiety, and post-traumatic stress disorder (PTSD). Existing mental health interventions are medicalised, primarily focusing on infant care. As a result, there is currently a lack of emotionally-focused interventions aimed at supporting parents after preterm birth. The current conceptual review proposes a compassion-focused framework to address the unique challenges faced by these parents. The paper aims to: (1) examine common experiences of parents of preterm-born infants, (2) explore the complex psychological processes that underpin these experiences, (3) present theoretical models that can be applied to understand parent’s psychological responses, (4) critically review existing interventions aimed at supporting parent mental health following preterm birth, (5) introduce a compassion-focused approach as a novel framework for support, (6) review existing compassion-based interventions aimed at perinatal populations, and (7) outline directions for future research. By integrating a compassion-focused approach, this paper aims to provide actionable insights to support parents’ mental health following preterm birth.

*Clinical Trial Number* not applicable.

## Introduction

The World Health Organisation ([WHO], 2023) defines preterm birth as a live birth occurring before 37 weeks of gestation. Globally, preterm birth accounted for 9.9% of live births in 2020, compared to 9.8% in 2010. Western countries, including the United States, Australia, and Europe, saw a slight reduction in the number of preterm births, decreasing from 1.13 million in 2010 to 1.04 million in 2020. However, this reduction was primarily driven by a decline in overall birth numbers rather than a specific decrease in the rate of preterm births (WHO, 2023). Thus, the occurrence of preterm birth has remained relatively steady across the developed world.

The lack of progress in reducing preterm birth rates can be attributed to factors such as the increasing use of assisted reproductive technologies, which are associated with an increase in multiple pregnancies and a 1.46 times greater risk of preterm birth relative to those who conceive spontaneously (Gorgui et al., 2020). Additionally, a societal shift towards delayed parenthood means that older childbearing age further compounds these risks (Fuchs et al., 2018). Though advances in neonatal care have improved survival outcomes for many preterm-born infants, preterm birth remains the leading cause of neonatal mortality worldwide, among children under five years of age, accounting for approximately 70% of infant deaths (Ananth et al., 2005; WHO, 2023). In addition to greater mortality rates, research has consistently shown that preterm birth increases the risk for longer-term adverse outcomes across physical, cognitive, and neurodevelopmental domains. For example, children born preterm face significantly greater risk for cerebral palsy, intellectual impairment, developmental delay, visual and hearing impairments, speech and language delays, and neurodevelopmental diagnoses (Eves et al., 2021; Sarda et al., 2021; Schieve et al., 2016; Ylijoki et al., 2024).

Parents play a critical role in shaping the developmental outcomes of these infants (the term “parents” will be used throughout this article, reflecting the current research landscape, which predominantly centres on the maternal experience, alongside emerging research of fathers. However, this terminology does not fully capture the diverse experiences of all caregivers or family structures; Larsson et al., 2022). Therefore, preterm birth is not merely a medical event affecting the health of the newborn—it can also have significant psychological implications for their parents. There are many unique challenges associated with caring for a preterm-born infant that can activate complex psychological processes and patterns of self-to-self relating, characterised by self-criticism and shame. These are well-established transdiagnostic processes that contribute to the development and maintenance of a range of mental health difficulties (Gilbert & Procter, 2006). Therefore, it is not surprising that parents report significantly worse mental health outcomes after preterm birth than a typical term birth, specifically on measures of postpartum depression (PPD), anxiety and post-traumatic stress disorder (PTSDDe Paula Eduardo et al., 2019; Gondwe et al., 2020; Malouf et al., 2022; Pace et al., 2016). Despite parents of preterm-born infants experiencing disproportionately worse mental health outcomes, there is a lack of emotionally-focused and accessible mental health support intervention options available to them. To date, interventions and support for this population of parents are often medically focused and predominantly prioritise infant care; however, a broader biopsychosocial approach is needed. The current conceptual article posits that a compassion-focused framework offers a promising avenue for supporting these parents. Taking this biopsychosocial approach, the current article will: (1) examine common experiences of parents of preterm-born infants, (2) explore the complex psychological processes that underpin these experiences, (3) present theoretical models that can be applied to understand parent’s psychological responses, (4) critically review existing interventions aimed at supporting the mental health of parents after preterm birth, (5) introduce a compassion-focused approach as a novel framework for support, (6) review existing compassion-based interventions aimed at perinatal populations, and (7) outline directions for future research.

## Preterm Birth Experiences

**Fig. 1:**
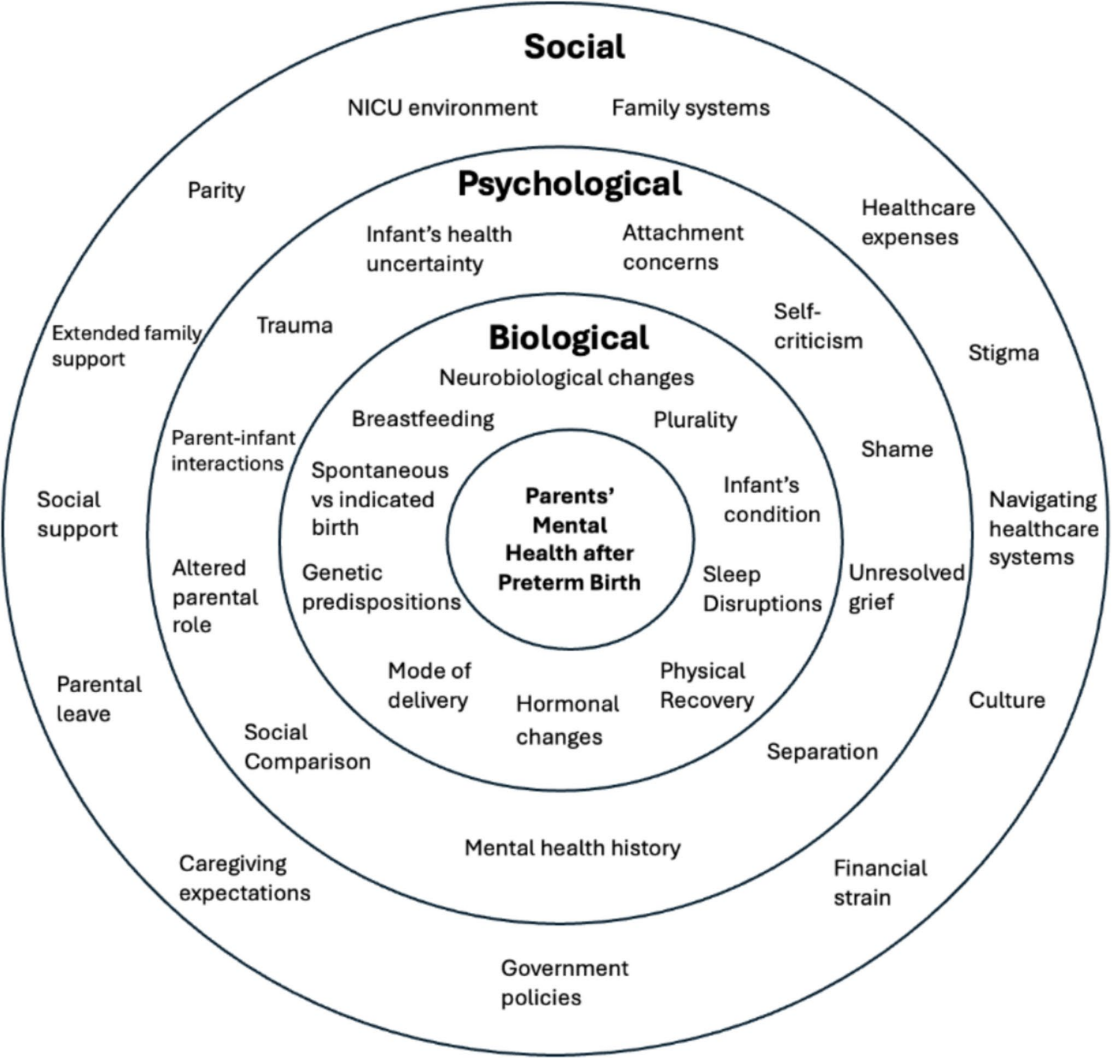
Biopsychosocial influences on the mental health of parents after preterm birth

Given the unique and complex nature of preterm birth, no two experiences are identical. As demonstrated in Fig. 1, parents’ experiences and vulnerability to mental health difficulties are shaped by a combination of biological, psychological and social factors.

### Mental Health History

A foundational factor that may shape how parents respond to preterm birth is their history of mental health difficulties. A history of mental health diagnoses is correlated with perinatal depression in women (Yang et al., 2022). Approximately 5% of women with no prior history of depression develop PPD, compared with 65% of those who were diagnosed with depression before or during pregnancy (Johansen et al., 2020). A history of mental health treatment is also associated with a higher risk of paternal depression in the early postpartum period (Nishimura & Ohashi, 2010).

### Spontaneous and Indicated Birth

Beyond pre-existing vulnerabilities, the experience of preterm birth may differ depending on whether preterm birth is indicated or spontaneous. Typically, many health behaviours (e.g. tobacco use, weight, nutrition, psychosocial factors) and pregnancy risks (e.g. chronic hypertension, hyperthyroidism, pregestational diabetes mellitus) are monitored across pregnancy, allowing clinicians to advise parents to prepare for a preterm birth to some extent, or plan an iatrogenic preterm birth, where labour is medically induced early (Behrman, 2007; Griggs et al., 2020; Gyamfi-Bannerman & Ananth, 2014). However, approximately 50% of preterm births are spontaneous, occurring without warning (Costello et al., 2023).

Anticipating a preterm birth allows parents to prepare for the challenges ahead but often introduces its own set of burdens. The uncertainty surrounding the infant’s health can lead to heightened stress and hypervigilance during pregnancy, with birthing parents monitoring for subtle changes in their bodies and adjusting their lives to minimise the risk of preterm delivery (White et al., 2019). These adjustments may include significant lifestyle changes, such as bed rest, which may require the parents to quit or suspend work and seek external help to fulfil professional and family responsibilities (Hung et al., 2021). Research demonstrates that women facing an indicated preterm birth experience substantially higher levels of stress relative to those with healthy pregnancies (Hung et al., 2021). Stress during the prenatal period can significantly increase the prevalence of developing PPD in women (Ding et al., 2023). On the other hand, spontaneous preterm birth leaves parents feeling unprepared—not only for the challenges ahead, but for the sudden shift in identity, highlighting that not only is the infant born preterm, but they are “preterm parents” (Blackburn & Harvey, 2019; Teti et al., 2005).

While it might be assumed that parents experiencing high-risk pregnancies would be better prepared for preterm birth, evidence suggests that whether preterm birth is indicated or spontaneous has little bearing on the emotional impact that follows. Qualitative research suggests that despite warnings of a potential preterm birth, parents remain hopeful for a healthy delivery, leaving parents to reconcile their expectations with the realities of preterm birth (Henderson et al., 2016; Lasiuk et al., 2013).

### Mode of Delivery

Research demonstrates that the mode of delivery, whether vaginal or caesarean section, has significant implications for maternal mental health (Dekel et al., 2019). Caesarean sections are associated with a higher risk of complications and prolonged physical pain and recovery time, contributing to increased mental health difficulties such as PTSD and depression relative to vaginal delivery (Grisbrook et al., 2022). This is particularly concerning as up to 36% of preterm births are delivered via caesarean section (Thanh et al., 2019).

### Infant’s Condition

The infant’s condition also significantly shapes parental experiences of preterm birth. Survival rates vary by gestational age and birth weight, with approximately 98% of infants born after 30 weeks surviving, compared with only 42–59% of those born before 24 weeks (Manuck et al., 2016; The American College of Obstetricians and Gynecologists, 2017). A dose–response relationship has been observed between the level of prematurity and depressive symptoms, with one study identifying higher depression scores for mothers of infants born at < 33 weeks’ gestation, compared with mothers of infants born at 33–35 weeks (Carter et al., 2005). This may be due to the increased likelihood of medical complications, prolonged stays in the Neonatal Intensive Care Unit (NICU) and uncertainty regarding the infant’s survival (Vigod et al., 2010).

### Parity

Following birth, parents must also adapt their existing lives to accommodate the newborn. Parity plays a significant role here, as primiparous and multiparous parents have distinct emotional responses to preterm birth. Research suggests that first-time mothers tend to experience adjustment difficulties immediately following birth. However, multiparous mothers are more likely to have more negative long-term outcomes (Gameiro et al., 2009). One study indicates that postpartum primiparous mothers report higher levels of psychological well-being compared with multiparous mothers (Bassi et al., 2017). Supporting this, a qualitative study by Arnold et al. (2013), found that some first-time parents of preterm-born infants felt less burdened by expectations of a “typical” birth. Without prior experiences shaping their expectations, they were better able to adapt to unfamiliar situations and rely on the NICU staff for guidance. In contrast, despite their prior child-rearing experience, multiparous parents face unique challenges in that they must not only reorganise their own lives for the new infant, but must also adjust existing parenting systems (Gameiro et al., 2009).

### Plurality

These challenges are even more pronounced for parents who give birth to multiples. Research indicates that parents of multiples are at an increased risk of depression, anxiety and parenting stress relative to parents of singletons (Wenze et al., 2015). Approximately 60% of twins and almost all higher-order multiples are born preterm (Australian Multiple Birth Association, 2022). In these cases, the challenges associated with preterm birth are significantly compounded (Findler et al., 2008).

#### NICU

Approximately 77% of preterm-born infants require care in the NICU, further shaping the parents’ experiences (Australian Institute of Health & Welfare, 2025). Mothers of preterm-born infants describe that one challenging aspect of their infant’s hospitalisation is the prolonged separation (Nyström & Axelsson, 2002). This separation begins the moment the infant is born, as the longed-for chance to hold their newborn is often thwarted by the need for urgent medical care. Evidence from Citter and Ghanouni (2021) found that only 38% of women who gave birth in the NICU were able to hold their babies within the first hour, compared with 92% of mothers of healthy infants, who were able to hold their newborns immediately and for as long as they wished. Prolonged separation may continue throughout the infant's stay in the NICU, as the infant’s vulnerability often necessitates that the NICU staff assume responsibility for even the most basic caregiving tasks (Lindberg & Öhrling, 2008). The first hours and days after birth are a critical period for interaction and bonding, which are essential for fostering early mother–child attachment and consolidating maternal confidence in the parenting role (Feldman et al., 1999). Therefore, disruptions during this time can interfere with this attachment process and cause them to feel insecure in their parenting abilities (Treherne et al., 2017; Woodward et al., 2014).

This separation is further exacerbated by the presence of medical equipment (Lantz & Ottosson, 2013). Parents of preterm-born infants often describe medical devices, such as incubators and ventilators, as creating a perceived barrier that separates them from their infant, limiting important bonding experiences, such as skin-to-skin care (Cervantes et al., 2011).

Beyond disrupting attachment, the artificial NICU can be a highly distressing environment for parents. They often report being confronted with ominous-sounding medical language (e.g., respiratory distress syndrome) and unfamiliar, alien-like equipment (White et al., 2019). Parents describe how the constant monitoring of their child and witnessing them undergo painful or invasive procedures, such as intubation and IV insertions, adds to their distress. In some cases, they may even be asked to assist medical staff in restraining their infant during these procedures, leading to feelings of powerlessness and a sense of betraying their child (Kantrowitz-Gordon et al., 2016).

The average length of hospital stay for term-born infants is approximately three days. In contrast, preterm-born infants typically remain hospitalised for about three times longer, with many requiring ongoing care for several weeks or even months (Lin et al., 2022; Ringborg et al., 2006). As a result, many parents of preterm-born infants are forced to return home without their newborn.

Ultimately, these experiences contribute to a perceived alteration to their parental role (Danerek & Dykes, 2008; Goutaudier et al., 2011; Lasiuk et al., 2013). Meta-analyses show this disruption to the parental role is among the most distressing aspects of mothers’ experiences of preterm birth (Caporali et al., 2020; Pal et al., 2024). Similarly, fathers of preterm-born infants report feeling distant from their parental role, identifying themselves as the “partner” of the mother, rather than the “parent” of the infant (Provenzi & Santoro, 2015).

In response to these challenges, there has been a global shift towards adopting family-inclusive care models such as Family-Centred Care (FCC), Family-Integrated Care (FIC), and Single-Family Rooms (SFR) to engage parents as active participants in their infant’s care. Systematic reviews and meta-analyses show that FCC improves parental participation, satisfaction and reduces distress during hospitalisation (Hodgson et al., 2025; Sabnis et al., 2019), while FIC reduces parental stress and anxiety (Moreno‐Sanz et al., 2025). SFR models also enhance parental presence, skin-to-skin contact, and lower NICU-related stress (Van Veenendaal et al., 2020). Although there is some evidence to suggest that FIC may reduce post-discharge PTSD, longitudinal research on how these NICU care models affect long-term parental mental health remains limited (Hodgson et al., 2025; Van Veenendaal et al., 2020).

### Transition from Hospital to Home

Transitioning home following discharge from the hospital can be stressful for any parent; however, this transition is often particularly challenging for parents of preterm-born infants, who may experience heightened anxiety and hypervigilance due to their infants’ increased vulnerability (Lakshmanan et al., 2019). Without the support of a 24/7 NICU care team, parents are suddenly faced with the challenge of managing their newborn’s complex needs on their own (Baraldi et al., 2020). As such, many parents of preterm-born infants report feeling unprepared for hospital discharge (Aydon et al., 2018; Trumello et al., 2018), particularly mothers with mental health difficulties who report higher levels of unpreparedness than those without (McGowen et al., 2017). For example, breastfeeding can be challenging due to the infant’s underdeveloped sucking and swallowing reflexes, weak muscles, latching difficulties, and breathing issues. Sleep disruption is another significant demand as preterm-born infants are often more unsettled and wake frequently during the night (Baumgartel & Facco, 2018; Haddad et al., 2019).

Typical parenting responsibilities like bathing and dressing can be challenging, as preterm-born infants’ small size and reliance on medical devices make it hard to find appropriate clothing and manage routine care (Danila et al., 2022). Furthermore, these infants often need medications and specialised medical equipment at home, requiring parents to administer treatments and monitor devices. Some mothers describe becoming dependent on these devices to monitor their child’s wellbeing (Granero-Molina et al., 2019). Frequent medical appointments, such as neurodevelopmental follow-up, can further overwhelm parents, who must juggle the logistics of transportation and managing medical equipment. This process is both physically and mentally taxing (Phillips-Pula et al., 2013).

There are a variety of outpatient support programs designed to support parents of preterm-born infants transition from hospital to home. These programs vary significantly in their structure, frequency and intensity, including public health visits, parental education sessions, providing parents with take-away information, 24-h telephone support and counselling (Hamer et al., 2023; Kim & Kim, 2024). While these programs have been shown to reduce hospital stay and re-admission rates as well as parent-infant interactions, findings related to parental outcomes remain mixed (Hamer et al., 2023). Some research suggests that these programs enhance parental autonomy and confidence (Schuetz Haemmerli et al., 2022). However, a recent systematic review and meta-analysis indicates that there is limited evidence examining the impact of these programs on parental mental health outcomes (Griffith et al., 2022; Hamer et al., 2023).

### Prolonged Trauma

Meta-analytic evidence across 35 studies (consisting of 2,708 mothers and 669 fathers) indicates that approximately 27% of parents of infants admitted to the NICU experience posttraumatic stress symptoms (PTSS) more than one year following birth (Malouf et al., 2022).

Even after an infant’s condition has stabilised and survival is no longer the primary concern, parents of preterm-born infants often continue to experience trauma. Ongoing developmental and health challenges can keep parents in a state of hypervigilance, with even minor changes in their child’s condition triggering distress. This heightened awareness may cause parents to relive the trauma of the NICU, particularly when delays in reaching milestones occur, or as new challenges emerge at school age (Treyvaud et al., 2014). Trauma can also be reactivated during healthcare appointments, where recounting medical history prompts emotional distress that may take days to subside (Dewan et al., 2023). Adding to these ongoing worries, physical reminders of the child’s early medical interventions also play a role in maintaining the trauma. Marks on the child’s body, such as scars from surgeries or other procedures, such as respiratory support, serve as constant reminders of the medical procedures their child endured in the NICU (Kantrowitz-Gordon et al., 2016).

As a result, many parents continue to experience trauma symptoms for months or even years postpartum. Notably, Barthel et al. (2020) found PTSS remained elevated for up to 5 years postpartum among mothers and fathers of preterm-born infants, compared with those of term-born infants.

This prolonged distress can contribute to Vulnerable Child Syndrome (VCS), in which parents, following their child’s illness, continue to perceive them as medically fragile or at risk, even when they are objectively healthy (Green & Solnit, 1964). VCS significantly impacts parent–child interactions and family dynamics, often leading to poorer parenting practices and overuse of healthcare services (Allen et al., 2004; Duncan & Caughy, 2009). As a result, children may experience poorer cognitive and behavioural outcomes (Chambers et al., 2011).

This trauma can potentially extend into future pregnancies, as a history of preterm birth is a well-established risk factor for subsequent preterm deliveries (Yang et al., 2016). There is even evidence to suggest that this trauma could potentially be passed from one generation to another through epigenetic mechanisms (Bohacek & Mansuy, 2015).

## Gender Differences in Parental Experiences

Mothers and fathers of preterm-born infants share several common experiences, including shock at the unexpected birth, high stress, difficulties bonding with the infant, and a sense of alienation in the NICU (Fegran et al., 2008; Hagen et al., 2016). However, there are distinct differences in their experiences following preterm birth. Mothers’ experiences are often more physically demanding, shaped by recovery from childbirth (e.g., post-delivery pain, lochia flow, breast engorgement), and the additional challenges of breastfeeding and milk expression (Kantrowitz-Gordon et al., 2016). Their attention is centred on caregiving and establishing an intimate relationship with the infant, reflecting a rapid transition from pregnancy to motherhood (Provenzi & Santoro, 2015).

There is significantly less research on fathers' experiences following preterm birth. However, available evidence suggests that fathers may feel excluded from primary caregiving tasks, as early interactions often centre on the mother-infant dyad (Adama et al., 2025; Stefana et al., 2018). In addition, they may often prioritise their partner and infant's wellbeing over their own (Hagen et al., 2016; Provenzi et al., 2016). Their caregiving often focuses on practical tasks and NICU-related activities, and early return to work can leave mothers to lead much of the hands-on care, delaying fathers’ transition into parenthood (Provenzi et al., 2016).

## Mental Health Outcomes

Parents who experience a preterm birth are more likely to develop mental health difficulties such as PPD, anxiety, and PTSD (Ponti et al., 2022). As such, mothers of preterm-born infants report higher rates of PPD of up to 40%, relative to 10–15% of mothers of full-term infants (Vigod et al., 2010). In addition, PTSD affects up to only 5.4% of mothers from general postpartum populations (Jenkins et al., 2024), however the prevalence is far higher among mothers of preterm-born infants, with rates reaching up to 71% (Gökçe İsbir et al., 2023). Mothers who give birth to a preterm-born infant have a 1.3 times increased risk of presenting to the emergency department for a mental health diagnosis within the first year postpartum (Calthorpe et al., 2021). This heightened vulnerability is alarming, as suicide is the most common cause of direct maternal death in the first 12 months following childbirth (Knight et al., 2023).

Fathers of preterm-born infants also experience significantly worse mental health outcomes. For example, 36% of fathers report depression following preterm birth, relative to 5% of fathers of term-born infants (Pace et al., 2016). Anxiety is also significantly higher for fathers after preterm birth, with rates of approximately 27%, compared with only 16% of fathers following term birth (Petersen & Quinlivan, 2021).

Notably, a meta-analysis of 79 studies identified a significant association of postpartum depression and anxiety between mothers and fathers of preterm-born infants. This co-occurrence of distress in families suggests that the mental health of both parents must be addressed simultaneously (Nguyen et al., 2023).

The mental health of parents of preterm-born infants can have significant negative implications for family dynamics, parent–child interactions, and the child's overall developmental outcomes (Treyvaud et al., 2014). In particular, maternal depression is associated with impairments in the infant's cognitive, behavioural, and psychomotor development by the age of one year (Kingston et al., 2012). This early disruption in infant development due to maternal depression can have lasting consequences, as studies show that maternal PPD is associated with depression in offspring at age 18 (Pearson et al., 2013).

## Complex Psychological Processes after Preterm Birth

Parenthood introduces profound physical, biological and psychological adaptation toward care-seeking and care-giving behaviour (Barba-Müller et al., 2019; Feldman, 2012; Wigert et al., 2006). During pregnancy, mothers construct rich mental representations of their unborn child, where they internally construct perceptions of their infant (Bowlby, 1980). These representations may include ideals about their infant's appearance, characteristics, and behaviour. Mental representations are most active between 4 and 7 months of gestation, with reduced activity in the 8th month. It is suggested that this decline is to prepare the parent for any potential disappointment and allow them to construct realistic expectations. Mothers with a healthy, low-risk birth show stable mental representations over the first year of life (Benoit et al., 1997). However, preterm birth often interrupts the typical progression of these mental representations, due to the unexpected nature of labour and reduced preparedness, leaving parents with unresolved or unadjusted expectations. For example, mothers report less acceptance of their infant’s appearance and/or characteristics and are more likely to have unrealistic and unsupported fears about their infant’s safety and survival (Trumello et al., 2018; Zeanah & Benoit, 1995). Mothers’ representations after preterm birth are characterised by confusion or disorientation, whereas fathers’ representations are more disengaged and marked by withdrawal (Tooten et al., 2014). Nevertheless, incongruencies between mental representations and the reality of preterm birth is associated with grief and difficulties adjusting (Agostini et al., 2009; Korja et al., 2009; Shah et al., 2011; Stern, 1991).

Approximately 32%—62% mothers of preterm-born infants report feelings of unresolved grief (Shah et al., 2011; Yaari et al., 2015). Additionally, in one study of 120 fathers of preterm-born infants, all fathers reported anticipatory grief reactions (Zamanzadeh et al., 2013). Coupled with the threat to their infant’s life, parents may experience a profound sense of loss, as their deeply internalised representations of birth and parental identity are shattered, even if the infant survives (Marvin & Pianta, 1996). Unlike traditional grief, which is typically associated with the loss of a person, the grief following preterm birth is tied to the loss of anticipated experiences and roles. Because society expects joy following the birth of an infant, the grief experienced by these parents is often disenfranchised (Golish & Powell, 2003). Failing to resolve this grief has mental health implications for parents; for example, unresolved grief has been linked to increased depression risk in mothers of infants with cerebral palsy (Krstić et al., 2015). In turn, parental mental health may also impact the resolution of this grief. Research suggests that higher maternal stress early in the postpartum period may predict a greater likelihood of remaining unresolved up to 18 months after birth. (Yaari et al., 2017).

## Intrapersonal Processes after Preterm Birth

Research indicates that parents of preterm-born infants report feelings of shame (Heidari et al., 2012; Jarašiūnaitė-Fedosejeva et al., 2024). Shame arises from negative self-evaluation, often linked with feelings of inferiority, and perceptions of not meeting societal or personal expectations (Gilbert, 2009). For example, mothers of preterm-born infants may feel as though they have failed in their ability to carry their infant to term, and for not meeting societal expectations of a “typical” pregnancy and childbirth. In postpartum populations, maternal shame is significantly associated with PPD (Dunford & Granger, 2017). A systematic review of 5 studies demonstrated that shame is associated with PPD and stress in mothers and stress, anxiety and depression in fathers (Caldwell et al., 2021). Specifically, mothers of preterm-born infants with PPD report higher levels of shame than those without PPD (Taburoğlu et al., 2024).

In response to shame, parents of preterm-born infants may engage in self-criticism. Self-criticism is a self-to-self relating style characterised by harsh self-evaluation (e.g., “*It is my fault they were born too early*”; Blatt, 2008; Zuroff et al., 2016). Self-criticism has implications for a range of mental health difficulties, particularly for maternal mental health (Gerhardt et al., 2024). There is a significant and positive association between self-criticism and PPD (Brassel et al., 2020), with inpatient mothers with PPD reporting significantly higher self-criticism than their non-depressed counterparts (Vliegen & Luyten, 2009). Specifically, in a descriptive study of 50 parents of preterm-born infants (43 mothers, 7 fathers), more than half of the sample used self-criticism as a coping mechanism (Mahmoud et al., 2022). Additionally, in qualitative study of 15 parents (14 mothers, 1 father), participants also frequently reported feelings of self-criticism after preterm birth (Voultsos et al., 2024). This is of concern, as research has identified a correlation between self-criticism in mothers of preterm-born infants and higher rates of postpartum emotional distress (Assal‐Zrike & Atzaba‐Poria, 2025).

Further to increasing the risk for mental health difficulties, shame can also impact support-seeking behaviours. Marsden et al. (2025) identified that mothers who experience shame related to their mental health symptoms are less likely to seek support driven by fears of not being perceived as a good or capable mother.

## Self-Compassion

Compassion can be defined as a response to perceiving suffering in oneself or others, coupled with a motivation to alleviate it (Gilbert, 2014). According to evolutionary perspectives, compassion stems from caregiving behaviours, which developed in species with fewer offspring and high parental investment. These behaviours enhanced the ability of parents to recognise distress in their offspring, to form strong attachment bonds necessary for survival, growth, and physiological regulation (Goetz et al., 2010).

Self-compassion is compassion that is directed inwards. It involves relating to oneself with care and support during suffering (Gilbert, 2014; Kirby, 2025; Neff, 2003). Research indicates that mothers of early childhood children who were born preterm report low levels of self-compassion (Fitzallen et al., 2024). Low levels of self-compassion have been associated with greater levels of depression and anxiety symptoms among perinatal women (Felder et al., 2016) and are also associated with more severe PTSD symptoms in individuals who have experienced traumatic events (Valdez & Lilly, 2016). In contrast, research has shown that greater self-compassion is linked to lower levels of depression, anxiety and stress in adult populations (MacBeth & Gumley, 2012) and, more specifically, parent populations (Jefferson et al., 2020).

## Theoretical Models to Understand Psychological Experience of Parents of Preterm-born Infants

### Self-Discrepancy Theory

According to Self-Discrepancy Theory, distress arises when there are perceived incompatibilities between one’s ideal, ought and actual selves (Higgins, 1987). Applying this to parents after preterm birth, the ideal self might be having a low risk and healthy infant. The ought self, in contrast, reflects perceived obligations and social expectations, such as meeting the standards of a “good parent”. However, parents are confronted with an actual self that feels inferior and distant from their parental role. Research demonstrates that discrepancies between the ideal and actual selves elicit feelings of shame and guilt (Liss et al., 2013) and are associated with depression and anxiety (Oh et al., 2023).

### Social Mentality Theory

According to Social Mentality Theory, individuals interpret their internal and external world through competitive or compassionate motivations. Social Mentality Theory can be used to explain how self-criticism and shame can be experienced in the parenting role (Kirby et al., 2019). For example, when confronted with a threat, anticipatory or sudden (e.g., preterm birth), a *competitive* motive orients a person to engage in social comparison to determine their relative rank to the threat (*e.g., “Am I inferior/superior to other parents”*). This can either be through upward social comparison, in which individuals compare themselves to those they perceive as better off, or through downward comparison, where they view their own situation as more favourable than that of others (Diel et al., 2021). Social Mentality Theory is a helpful framework for understanding the mechanisms contributing to parents’ complex experiences following preterm birth. For instance, parents may make comparisons to other parents of preterm-born infants who are further along in their journey, which may provide a source of hope. Comparatively, they make comparisons to parents of healthy infants, which may contribute to worry about their child’s development or not meeting societal expectations of their caregiving role (Lakshmanan et al., 2019), leading to increased self-criticism and shame (Sturman, 2011).

## Existing Interventions to Support the Mental Health of Parents after Preterm Birth

Several reviews have examined the effectiveness of interventions aimed at supporting the mental health of parents after preterm birth (Benzies et al., 2013; Chan & Shorey, 2024; Dewi et al., 2025; Laccetta et al., 2023; Mendelson et al., 2017; Puthussery et al., 2018; Seiiedi-Biarag et al., 2020; Zhang et al., 2021). While findings show positive effects across measures of maternal mental health (depression, anxiety and PTSD), parental self-efficacy, parental confidence and parent-infant interactions, there is significant heterogeneity between intervention targets, delivery methods and outcome measures.

While some interventions are assessed only during the NICU stay, others evaluate longitudinal outcomes up to 9 years postpartum (Landsem et al., 2014). Notably, most studies only include mothers (Chan & Shorey, 2024; Dewi et al., 2025).

Existing interventions address a broad spectrum of approaches. The most common interventions prioritise mother-infant interactions, such as the Transactional Theory of Development (Kaaresen et al., 2006; Landsem et al., 2014; Newnham et al., 2009; Ravn et al., 2012), Triadic Parent-Infant Relationship Therapy (Castel et al., 2016), educational interventions (Collette et al., 2023; Franck et al., 2011; Salehnezhad et al., 2022) and Kangaroo care (Holditch-Davis et al., 2014). Other methods include Cognitive Behavioural Therapy (Bernard et al., 2011; Hagan et al., 2004; Shaw et al., 2014; Silverstein et al., 2011), expressive writing (Horsch et al., 2016), mindfulness-based stress reduction (Izadi et al., 2022), and trauma-focused interventions (Jotzo & Poets, 2005; Shaw et al., 2013). While there are some online modalities (Bahmanpour et al., 2023; Benzies et al., 2020; Collette et al., 2023; Garfield et al., 2016; Hägi-Pedersen et al., 2022; Huang et al., 2024), the primary delivery method for these interventions is in-person sessions, ranging from three sessions (Bernard et al., 2011) to 22 sessions (Castel et al., 2016).

Studies evaluating mental health interventions for parents following preterm birth assess a diverse range of outcomes. The most measured maternal mental health outcomes include depression, anxiety and PTSD. Parental outcomes frequently assessed are parenting stress, self-efficacy, confidence and competence. Other key areas of measurement include parent-infant interactions, bonding, and infant developmental outcomes.

Existing mental health interventions for parents after preterm birth vary widely, typically focusing on improving infant care, parent-infant interactions and supporting the transition into caregiving roles. While these areas are important, it is not sufficient in addressing the emotional experiences of parents of preterm-born infants. Recent evidence highlights the need for addressing transdiagnostic processes such as self-criticism, shame and self-compassion in interventions targeting the mental health of these parents (Assal‐Zrike & Atzaba‐Poria, 2025; Fitzallen et al., 2024; Jarašiūnaitė-Fedosejeva et al., 2024).

Current interventions are also primarily conducted in person. In-person interventions pose limitations regarding accessibility, particularly for parents who may be dealing with the stigma, time constraints and logistical challenges of preterm caregiving. For example, preterm birth rates are higher in regional and remote areas compared with metropolitan cities (Bizuayehu et al., 2023), with First Nations women in Australia facing some of the highest preterm birth rates globally (Brown et al., 2024). However, their access to specialised healthcare, such as mental health support, is often limited.

Access issues such as these contribute to consistently low attendance for perinatal mental health care. For example, in an Australian context, of only 37.1% of women who are offered mental health support, only 36.6% accepted, and only 40.0% of those attended the appointment. Engagement was dependent on a lack of time, childcare and encouragement by healthcare providers and family (Ayres et al., 2019).

There also remains a significant gap in capturing the diverse experiences of parents after preterm birth. Research in this area has predominantly focused on mothers. While understanding the maternal experience is crucial, it is not all-encompassing and may reinforce the stereotype that mothers are sole or primary parents. The experiences of preterm birth are not homogenous, yet many perspectives remain underexplored. Recent efforts have endeavoured to include fathers' experiences and their mental health outcomes (Agostini et al., 2022; Ionio et al., 2016; Mackley et al., 2010; Merritt, 2021; Petersen & Quinlivan, 2021; Provenzi & Santoro, 2015), marking an important step toward broader representation. However, they remain largely underrepresented in samples. Diverse family structures, cultural backgrounds and their members have also not been adequately prioritised or represented in research, despite many of these populations facing a higher incidence of preterm birth (Chakraborty et al., 2024; Hamilton et al., 2023) and postpartum mental health difficulties (Adler et al., 2023; Onyewuenyi et al., 2023). These underrepresented groups encounter additional and distinct challenges related to societal expectations, caregiving demands, and mental health, which can further intensify the trauma associated with preterm birth (Attanasio & Hardeman, 2019; Davis, 2019; Karbeah et al., 2022; Klittmark et al., 2023; Milford et al., 2023). Expanding research to include these varied experiences is imperative to developing more inclusive, representative, and effective support for all parents of preterm-born infants.

## Compassion Focused Therapy (CFT)

CFT is a psychotherapeutic approach that integrates principles from evolutionary, neuroscience and Buddhist practices to assist clients in developing self-compassion, compassion to others and an openness to compassion from others in response to adversity or threatening situations (Gilbert, 2014). CFT interventions have shown efficacy in a range of populations, particularly where self-criticism and shame are the underlying processes of mental health difficulties (Boersma et al., 2015; Wakelin et al., 2022). Systematic reviews have demonstrated CFT’s effectiveness as a treatment for a range of mental health conditions (Craig et al., 2020; Millard et al., 2023; Petrocchi et al., 2024).

CFT was built on findings suggesting that there are three main emotional regulation systems which interact: the threat system that detects and responds to threat (e.g. fight, flight or freeze), the drive system that activates motivation and reward-seeking behaviours, and the soothing system which promotes affiliative behaviours. For example, a situation involving defensive fighting will activate the threat systems, while experiences of bonding tend to engage the soothing system. In parents of preterm-born infants, the threat system is often overactivated, triggering feelings of self-criticism and shame, while the soothing system is often underutilised. This imbalance may disrupt parents' abilities to experience affiliative emotions, which are essential for parent-infant attachment (Thirkettle et al., 2024). CFT offers a transdiagnostic intervention that directly targets these self-relating processes by strengthening the soothing system through cultivating self-compassion and affiliative emotions.

Therefore, if compassion evolved from caregiving behaviours rooted in high parental investment strategies, and if a primary shift for new parenthood is towards care-seeking and caregiving motives, then CFT, which specifically cultivates compassionate, caregiving motivations, may serve as an effective tool for parents in the postpartum period. CFT has been suggested to create positive shifts in how mothers relate to themselves and their infants, even in cases of significant bonding disruption (Cree, 2010).

## CFT for Parents after Preterm Birth

Given the unique psychological challenges faced by parents of preterm-born infants, marked by self-criticism and shame, compassion-based interventions may offer a particularly suitable approach for supporting their mental health. O’Boyle-Finnegan et al. (2022) provides preliminary evidence that self-compassion could help to alleviate depression, anxiety, and adjustment difficulties in parents of preterm-born infants. Similarly, Fitzallen et al. (2024) identified that psychosocial well-being was strongly associated with self-compassion for mothers of preterm-born children.

Evidence suggests that compassion-based interventions also show promise in improving the well-being of parents of children with complex care needs, such as those caring for children with autism (Neff & Faso, 2015; Torbet et al., 2019) and developmental disabilities (Ahmed & Raj, 2023), which are common outcomes for children born preterm.

## Compassion-Based Interventions Aimed at the Perinatal Period

While there are currently no compassion-based interventions designed specifically for parents of preterm-born infants, research conducted in perinatal periods (pregnancy to two years postpartum) suggests that CFT is an effective option for reducing mental health difficulties in parents (Fernandes et al., 2022). A recent systematic review of five studies, including 1,000 mothers, provided preliminary support for CFT in mothers with subclinical mental health symptoms, demonstrating significant reductions in self-criticism and shame, along with significant improvements in breastfeeding satisfaction, mother-infant attachment and self-compassion (Millard & Wittkowski, 2023).

Two recent studies have examined the efficacy of CFT groups in perinatal populations. Thirkettle et al. (2024) evaluated an 8-session, online perinatal CFT group with 30 participants. Significant reductions were found for self-criticism and psychological distress, as well as significant improvement in self-reassurance. Lawrence et al. (2024) also evaluated an online perinatal CFT group, obtaining data from 114 participants. Significant improvements were found for all measures, self-criticism, self-compassion, mental health, parent-infant bonding and personalised goals. This suggests that CFT may serve as an effective intervention for perinatal women experiencing significant mental health difficulties.

Several RCTs have also investigated CFT interventions in the perinatal period. These studies span diverse countries, including Australia, New Zealand, the UK, the US, Spain, Portugal, and India, demonstrating a broad international interest in compassion-based interventions. Participants were typically recruited from the second or third trimester of pregnancy (Guo et al., 2020) to up to 24 months postpartum (Lennard et al., 2021; Mitchell et al., 2018). Key mental health outcomes targeted in these studies were depression (Fonseca et al., 2019; Gammer et al., 2020; Guo et al., 2020; Kelman et al., 2018), anxiety, (Gammer et al., 2020; Guo et al., 2020; Kelman et al., 2018; Lennard et al., 2021) and stress (Gammer et al., 2020; Lennard et al., 2021; Mitchell et al., 2018). Secondary outcomes included self-criticism, shame, psychological flexibility, and parenting satisfaction, with some studies also exploring maternal well-being and self-regulation (e.g., Fonseca et al., 2019). Most were waitlist controls, although one study compared CMT with a CBT control group (Kelman et al., 2018).

## Future Research

### Adapting CFT for Parents after Preterm Birth

Parents of preterm-born infants experience heightened levels of self-criticism and shame, which in turn increase their vulnerability to mental health difficulties. Compassion-based interventions were developed to directly target these issues, and interventions examined in the postpartum period provide preliminary support for their application. However, there are currently no interventions that specifically evaluate the efficacy of compassion-based interventions within this population. Important considerations on applying such an approach to parents of preterm-born infants are 1)intensity of the intervention, 2) delivery modality, and 3) making the intervention directly relevant to the lived experiences of parents, as opposed to a generic program for all parents.

Ultimately, a range of intervention options will be needed to support parents, as some will be experiencing significant mental health challenges, such as PPD or PTSD, where high-intensity support would be recommended. However, many parents do not meet clinical thresholds for disorders yet experience shame and self-criticism. Research suggests that mothers frequently turn to the internet for parenting and health information, particularly during times of uncertainty and stress (Moon et al., 2019). These resources are often used in combination with traditional therapy methods to supplement professional advice (Baumann et al., 2020; Kemp et al., 2024). Thus, an easy-to-deliver and accessible intervention, such as an online self-directed or app-based program, would be a likely appealing modality option.

Importantly, parents of preterm-born infants are not a homogenous group. Mothers, fathers, single parents, non-birthing parents and extended family members may all be involved in caregiving roles, each facing distinct challenges. As such, modality and intensity of delivery should not only be tailored to clinical need, but also to the caregiving context and identity.

A compassion-based intervention may target the significant and often traumatic event of preterm birth, highlighting how unexpected, life-threatening experiences can trigger intense emotions such as fear, anger and sadness. A core approach to CFT interventions is to reduce shame around these emotional responses, validate parents’ experiences, and introduce self-compassion to strengthen their soothing system. This may help to regulate the threat and distress associated with caring for a preterm-born infant. Such an approach was used in a brief 2-h seminar for parents with high levels of self-criticism, which significantly reduced self-criticism and increased self-compassion (Kirby et al., 2023). However, for such an intervention to be truly effective and accessible, a key recommendation is to engage in a co-design process with parents. Co-design involves collaborating directly with stakeholders to incorporate their lived experiences, preferences and needs into intervention development (Steen, 2013). For example, including visual elements, such as images of infants with medical equipment, may be beneficial for this population, yet this needs to be heard directly from the parents themselves. Moreover, an iterative feedback process would be a crucial component for developing such an intervention, as it empowers participants by involving them in every stage of intervention development, from initial idea conceptualisation to prototyping and refinement (Sanders & Stappers, 2008). This method is particularly beneficial for preterm parents, given that they face highly unique challenges that generic interventions may fail to address.

## Conclusion

Parents of preterm-born infants face many unique challenges that can trigger feelings of self-criticism, shame and reduced self-compassion. As a result, they often report worse mental health outcomes relative to parents of term-born infants. However, there is a lack of emotionally-focused interventions for parents after preterm birth. Compassion-based interventions may be particularly beneficial for this population as they specifically target self-criticism, shame and self-compassion as transdiagnostic factors that underpin a variety of mental health difficulties. It is recommended that future research investigate the effectiveness of compassion-based interventions for parents of preterm-born infants and incorporate co-design methods to ensure that these interventions are tailored to their unique experiences and inclusive of a diverse range of parents.

## Data Availability

No datasets were generated or analysed during the current study.
